# Supplementary material for: Hybrid Models and Biological Model Reduction with PyDSTool
Source: PLoS Comput Biol. 2012 Aug 9;8(8):e1002628. doi: 10.1371/journal.pcbi.1002628 (PMC3415397; doi:10.1371/journal.pcbi.1002628)
Supplement: Text S4 — Complete source code for the PyDSTool package (version 0.88.120504). Includes API documentation and help files linking to web pages. This file is identical to the current public release on Sourceforge.net. (ZIP) [file pcbi.1002628.s004.zip › PyDSTool/html/PyDSTool.Variable'-module.html]

xml version="1.0" encoding="ascii"?


PyDSTool.Variable'


| Home | Trees | Indices | Help | PyDSTool |
| --- | --- | --- | --- | --- |

Package PyDSTool ::
Module Variable'

[hide private]

[frames] | no frames]

# Module Variable'

source code

Variable is a one-dimensional discrete and continuous real variable
class.

Robert Clewley, July 2005


|  |  |
| --- | --- |
| Classes | [hide private] |

HybridVariable  
Mimics part of the API of a non-hybrid variable.

OutputFn  
One-dimensional function wrapper.

VarDiagnostics

Variable  
One-dimensional discrete and continuous real variable class.


|  |  |
| --- | --- |
| Functions | [hide private] |

|  |  |
| --- | --- |
| iscontinuous(var)  Determine if variable is continuously defined on its input and output domains. | source code |

|  |  |
| --- | --- |
| isdiscrete(var)  Determine if variable is discretely defined on its input and output domains. | source code |

|  |  |
| --- | --- |
| isinputcts(obj) | source code |

|  |  |
| --- | --- |
| isinputdiscrete(var) | source code |

|  |  |
| --- | --- |
| isoutputcts(var) | source code |

|  |  |
| --- | --- |
| isoutputdiscrete(obj) | source code |

|  |  |
| --- | --- |
| numeric\_to\_vars(vals, coordnames, indepvar=None, indepvarname=`'``t``'`, indepdomain=None, all\_types\_float=True, discrete=True, abseps=None, labels=None)  Utility to convert numeric types to a dictionary of Variables. | source code |

|  |  |
| --- | --- |
| pointset\_to\_vars(pts, discrete=True)  Utility to convert Pointset to a dictionary of Variables. | source code |


|  |  |
| --- | --- |
| Variables | [hide private] |

API = `API_class()`

Continuous = `Continuous Domain`

Discrete = `Discrete Domain`

Inf = `inf`

LargestInt32 = `2147483647`

NaN = `nan`

\_1DimplicitSolveMethods = `['newton', 'bisect', 'steffe']`

\_all\_complex = `(<type 'complex'>, <type 'numpy.complexfloating...`

\_all\_float = `(<type 'float'>, <type 'numpy.floating'>, <type '...`

\_all\_int = `(<type 'int'>, <type 'numpy.integer'>, <type 'numpy...`

\_all\_numpy\_complex = `(<type 'numpy.complex128'>, <type 'numpy....`

\_all\_numpy\_float = `(<type 'numpy.float64'>, <type 'numpy.float...`

\_all\_numpy\_int = `(<type 'numpy.int32'>, <type 'numpy.int32'>, ...`

\_complex\_types = `(<type 'complex'>, <type 'numpy.complexfloati...`

\_float\_types = `(<type 'float'>, <type 'numpy.floating'>)`

\_implicitSolveMethods = `['newton', 'bisect', 'steffe', 'fsolve']`

\_int\_types = `(<type 'int'>, <type 'numpy.integer'>)`

\_num\_equivtype = `{<type 'float'>: <type 'numpy.float64'>, <typ...`

\_num\_maxmin = `{<type 'numpy.int32'>: [-2147483648, 2147483647]...`

\_num\_name2equivtypes = `{'float': (<type 'float'>, <type 'numpy...`

\_num\_name2type = `{'float': <type 'numpy.float64'>, 'int': <typ...`

\_num\_type2name = `{<type 'float'>: 'float', <type 'int'>: 'int'...`

\_num\_types = `(<type 'float'>, <type 'int'>, <type 'numpy.float...`

\_pytypefromtype = `{<type 'numpy.int32'>: <type 'int'>, <type '...`

\_real\_types = `(<type 'int'>, <type 'numpy.integer'>, <type 'fl...`

\_seq\_types = `(<type 'list'>, <type 'tuple'>, <type 'numpy.ndar...`

contained = `contained`

isfinite = `<ufunc 'isfinite'>`

notcontained = `notcontained`

null\_predicate = `null_predicate_class(None)`

targetLangs = `['c', 'python', 'matlab']`

uncertain = `uncertain`


|  |  |
| --- | --- |
| Function Details | [hide private] |

|  |  |
| --- | --- |
| numeric\_to\_vars(vals, coordnames, indepvar=None, indepvarname=`'``t``'`, indepdomain=None, all\_types\_float=True, discrete=True, abseps=None, labels=None) | source code |

Utility to convert numeric types to a dictionary of Variables. If
discrete option set to True (default is False) then the Variables will be
linearly interpolated within their domain.

|  |  |
| --- | --- |
| pointset\_to\_vars(pts, discrete=True) | source code |

Utility to convert Pointset to a dictionary of Variables. If discrete
option set to False (default is True) then the Variables will be linearly
interpolated within their domain.

Any labels in the pointset will be preserved in the Variables in case
of their re-extraction using the getDataPoints method.

  


|  |  |
| --- | --- |
| Variables Details | [hide private] |

### \_all\_complex

Value:
:   ```
    (<type 'complex'>,
     <type 'numpy.complexfloating'>,
     <type 'numpy.complex128'>,
     <type 'numpy.complex64'>,
     <type 'numpy.complex128'>)
    ```

### \_all\_float

Value:
:   ```
    (<type 'float'>,
     <type 'numpy.floating'>,
     <type 'numpy.float64'>,
     <type 'numpy.float32'>,
     <type 'numpy.float64'>)
    ```

### \_all\_int

Value:
:   ```
    (<type 'int'>,
     <type 'numpy.integer'>,
     <type 'numpy.int32'>,
     <type 'numpy.int32'>,
     <type 'numpy.int8'>,
     <type 'numpy.int16'>,
     <type 'numpy.int32'>,
     <type 'numpy.int64'>)
    ```

### \_all\_numpy\_complex

Value:
:   ```
    (<type 'numpy.complex128'>,
     <type 'numpy.complex64'>,
     <type 'numpy.complex128'>)
    ```

### \_all\_numpy\_float

Value:
:   ```
    (<type 'numpy.float64'>,
     <type 'numpy.float32'>,
     <type 'numpy.float64'>)
    ```

### \_all\_numpy\_int

Value:
:   ```
    (<type 'numpy.int32'>,
     <type 'numpy.int32'>,
     <type 'numpy.int8'>,
     <type 'numpy.int16'>,
     <type 'numpy.int32'>,
     <type 'numpy.int64'>)
    ```

### \_complex\_types

Value:
:   ```
    (<type 'complex'>, <type 'numpy.complexfloating'>)
    ```

### \_num\_equivtype

Value:
:   ```
    {<type 'float'>: <type 'numpy.float64'>,
     <type 'int'>: <type 'numpy.int32'>,
     <type 'numpy.integer'>: <type 'numpy.int32'>,
     <type 'numpy.floating'>: <type 'numpy.float64'>,
     <type 'numpy.int8'>: <type 'numpy.int32'>,
     <type 'numpy.int16'>: <type 'numpy.int32'>,
     <type 'numpy.int32'>: <type 'numpy.int32'>,
     <type 'numpy.int32'>: <type 'numpy.int32'>,
    ...
    ```

### \_num\_maxmin

Value:
:   ```
    {<type 'numpy.int32'>: [-2147483648, 2147483647],
     <type 'numpy.float64'>: [-inf, inf]}
    ```

### \_num\_name2equivtypes

Value:
:   ```
    {'float': (<type 'float'>,
               <type 'numpy.floating'>,
               <type 'numpy.float64'>,
               <type 'numpy.float32'>,
               <type 'numpy.float64'>),
     'int': (<type 'int'>,
             <type 'numpy.integer'>,
             <type 'numpy.int32'>,
    ...
    ```

### \_num\_name2type

Value:
:   ```
    {'float': <type 'numpy.float64'>, 'int': <type 'numpy.int32'>}
    ```

### \_num\_type2name

Value:
:   ```
    {<type 'float'>: 'float',
     <type 'int'>: 'int',
     <type 'numpy.integer'>: 'int',
     <type 'numpy.floating'>: 'float',
     <type 'numpy.int8'>: 'int',
     <type 'numpy.int16'>: 'int',
     <type 'numpy.int32'>: 'int',
     <type 'numpy.int32'>: 'int',
    ...
    ```

### \_num\_types

Value:
:   ```
    (<type 'float'>,
     <type 'int'>,
     <type 'numpy.floating'>,
     <type 'numpy.integer'>)
    ```

### \_pytypefromtype

Value:
:   ```
    {<type 'numpy.int32'>: <type 'int'>,
     <type 'numpy.float64'>: <type 'float'>}
    ```

### \_real\_types

Value:
:   ```
    (<type 'int'>,
     <type 'numpy.integer'>,
     <type 'float'>,
     <type 'numpy.floating'>)
    ```

### \_seq\_types

Value:
:   ```
    (<type 'list'>, <type 'tuple'>, <type 'numpy.ndarray'>)
    ```

  


| Home | Trees | Indices | Help | PyDSTool |
| --- | --- | --- | --- | --- |

|  |  |
| --- | --- |
| Generated by Epydoc 3.0.1 on Fri May 4 15:24:05 2012 | http://epydoc.sourceforge.net |
